# Plant associated protists—Untapped promising candidates for agrifood tools

**DOI:** 10.1111/1462-2920.16303

**Published:** 2022-12-12

**Authors:** Bao‐Anh Thi Nguyen, Kenneth Dumack, Pankaj Trivedi, Zahra Islam, Hang‐Wei Hu

**Affiliations:** ^1^ School of Agriculture and Food, Faculty of Veterinary and Agricultural Sciences The University of Melbourne Parkville Victoria Australia; ^2^ Terrestrial Ecology Institute of Zoology, University of Cologne Köln Germany; ^3^ Microbiome Network and Department of Agricultural Biology Colorado State University Fort Collins Colorado USA; ^4^ ARC Hub for Smart Fertilisers The University of Melbourne Parkville Victoria Australia

## Abstract

The importance of host‐associated microorganisms and their biotic interactions for plant health and performance has been increasingly acknowledged. Protists, main predators and regulators of bacteria and fungi, are abundant and ubiquitous eukaryotes in terrestrial ecosystems. Protists are considered to benefit plant health and performance, but the community structure and functions of plant‐associated protists remain surprisingly underexplored. Harnessing plant‐associated protists and other microbes can potentially enhance plant health and productivity and sustain healthy food and agriculture systems. In this review, we summarize the knowledge of multifunctionality of protists and their interactions with other microbes in plant hosts, and propose a future framework to study plant‐associated protists and utilize protists as agrifood tools for benefiting agricultural production.

## INTRODUCTION

Living plants are hosts of a complex microbiome, comprising of bacteria, fungi, archaea, protists and viruses that internally and externally colonize plant tissues (Hassani et al., 2018; Sapp et al., 2018; Trivedi et al., 2020). These beneficial, neutral and pathogenic plant‐associated microorganisms can significantly influence plant health and performance. The plant hosts and their associated microbiomes are suggested to form a ‘holobiont’, where complex plant–microbe interactions play crucial roles in regulating and promoting plant growth, biogeochemical cycling, nutrient acquisition, fitness and protection, stress tolerance and disease suppression (Hassani et al., 2018; Liu et al., 2019). Plant associated microbiota, in some cases, even contribute more to plant protection and stress resistance than the defensive capacity of plant hosts (Hubbard et al., 2019). A holistic microbiome perspective to decipher the mechanisms that govern the assembly, interactions and functions of plant‐associated microbiota, therefore, is a prerequisite to facilitate translational research and develop microbiome‐based tools to enhance plant productivity and agricultural sustainability.

A panoramic view of the plant microbiota cannot be complete without considering protists as a pivotal component. Bacteria dominate the plant microbiota, followed by fungi, while protists and other organisms (e.g., archaea, nematodes and other soil invertebrates) are less abundant, but they were shown to be crucial in plant health and performance (Leach et al., 2017; Chen et al., 2021). Bacteria and fungi in the rhizosphere are enriched by carbon sources stemming from root exudates of plants (via bottom‐up control), however, they are major microbial prey for protists and thus subject to top‐down control by protist consumers. Protists, representing the vast diversity of unicellular eukaryotes, function as consumers (main predators of bacteria, fungi and small animals), primary producers (important carbon fixers via photosynthesis), plant and animal parasites, and decomposers (Geisen et al., 2018). The contributions of protists to nutrient input, organic matter decomposition and plant health have been previously reported (Bonkowski, 2004; Xiong et al., 2020; Geisen et al., 2021). Nonetheless, plant‐associated protists and their functions for plant hosts, compared to bacteria and fungi, have been largely underestimated (Gao et al., 2019; Trivedi et al., 2020). While the importance of plant beneficial microorganisms as promising agrifood tools to improve crop production and agricultural sustainability has been increasingly recognized (Chen et al., 2021; Hu et al., 2022), the plant–protist–microbe interactions in the above‐ and below‐ground systems are not well understood.

## POTENTIAL FUNCTIONS AND INTERACTIONS OF PROTISTS IN THE PLANT–SOIL–MICROBE NETWORK

**FIGURE 1:**
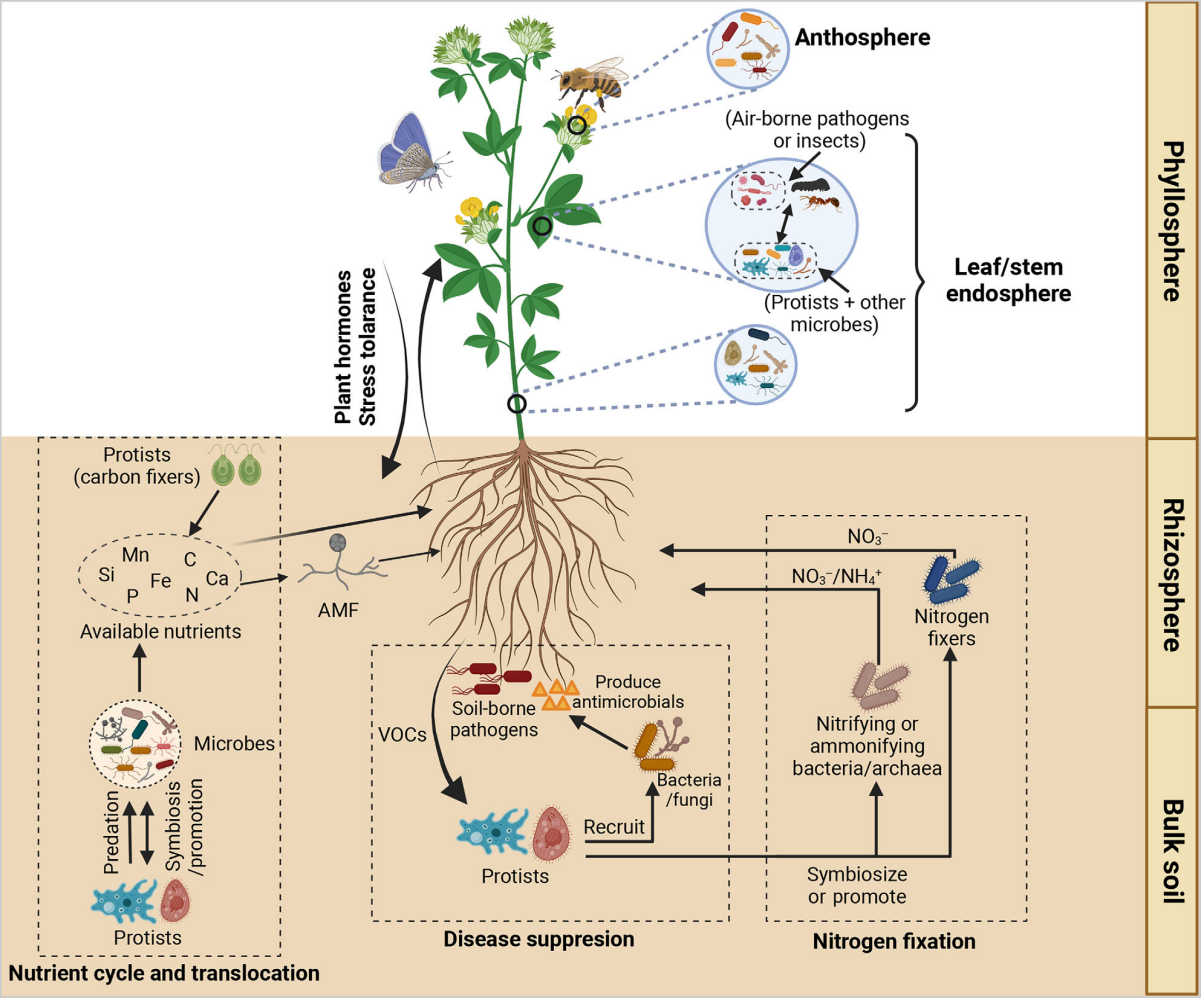
Functions and interactions of protists within the plant‐associated microbiota in different plant compartments (phylloshere, anthosphere, leaf, stem and root endosphere, rhizosphere and bulk soil). (i) Through predation or symbiosis, protists interact with bacteria, fungi (especially, arbuscular mycorrhiza fungi (AMF)) and other microbes (e.g., archaea) in cycling, uptake and/or translocation of essential nutrients (e.g., nitrogen, phosphorous, carbon, silicon, calcium, magnesium and iron) for plants and soil organisms in rhizosphere and bulk soils. Protists can also enhance nutrient input as carbon fixers or through releasing nutrients after the prey consumption; (ii) protists have the potential to form symbiotic or facilitative relationships with nitrifying and ammonifying bacteria or archaea in nitrogen fixation in plant rhizosphere; (iii) the predation of protists can trigger the antimicrobial production of bacteria or fungi, inhibiting the infection of air‐borne or soil‐borne pathogens or pests in phyllosphere and rhizosphere; (iv) protists may also enhance plant hormone and stress tolerance by directly interacting with plant hosts or stimulating plant‐beneficial traits of microbes.

Although protists have the great potential to improve nutrition, suppress pathogens, promote plant growth, and function as bioindicators for plant health (Bonkowski, 2004; Xiong et al., 2020), protists colonizing inside plant tissues remain vastly untapped. Most studies on plant‐associated protists have focused on plant pathogenic or parasitic protists causing plant diseases (Dumack & Bonkowski, 2021) or the belowground protist community particularly in the rhizosphere (Fiore‐Donno et al., 2022). Bacteria and fungi are well‐characterized plant microbiome components with distinct community compositions across different compartments (e.g., phyllosphere, anthosphere, leaf and root endospheres, rhizosphere, and bulk soils) (Liu et al., 2019; Trivedi et al., 2020; Sun et al., 2021b). Given the selective feeding preference of different protist groups for bacteria and fungi (Dumack et al., 2020), plant compartments at different developmental stages may harbour distinct taxonomic and functional diversity, community structure and functions of protists. In this article, we discuss known functions of protists and propose their potential roles and activities in different compartments of plants (Figure 1; Table 1).

**TABLE 1:** Known beneficial functions of plant‐associated protists in different plants

Plants	Plant compartments	Protist species	Functions of protists	References
Banana plants	Rhizosphere (roots and soil)	*Cercomonas lenta* strain ECO‐P‐01	Plant disease suppression against *Fusarium* wilt; Plant yield	(Guo et al., 2022)
Faba bean (*Vicia faba*) seedlings	Rhizosphere (plant roots)	*Rosculus terrestris* S14D1; *Bodomorpha* sp. C10D3; *Cercomonas lenta* C5D5	Plant disease suppression against *Fusarium* rot; Root length and biomass improvement	(Bahroun et al., 2021)
Cucumber	Rhizosphere	Protist community	Indicators and determinants of plant yield and disease suppression	(Guo et al., 2021)
Tomato	Rhizosphere	Protist community	Determinants of plant yield and disease suppression	(Xiong et al., 2020)
Wheat	Phyllosphere; Rhizosphere	Consumers	Plant uptake and translocation of inorganic nitrogen	(Clarholm, 1985)
Wheat	Phyllosphere and Rhizosphere	*Acanthamoeba castellanii*	Plant shoot/root biomass; Nitrogen translocation within root system	(Henkes et al., 2018)
*Arabidopsis thaliana*	Rhizosphere (plant roots)	*Acanthamoeba castellanii*	Plant uptake of carbon; Plant uptake of nitrogen; Plant growth (root and shoot biomass) and seed production	(Krome et al., 2009)
Ryegrass seedling	Rhizosphere	Naked amoebae, flagellates and ciliates	Nitrogen availability; Plant growth	(Bonkowski et al., 2000)
Spruce seedlings	Rhizosphere (root systems); phyllosphere (stems and needles)	*Acanthamoeba* sp.	Plant phosphorus and calcium uptake and translocation; Biomass of plant compartments	(Bonkowski et al., 2001)
Rice plants (*Oryza sativa* L.)	Rhizosphere	*Acanthamoeba castellanii*	Root growth and shoot biomass; Plant carbon, phosphorus, magnesium, and calcium uptake	(Herdler et al., 2008)
*Arabidopsis thaliana*	Rhizosphere	*Acanthamoeba castellanii*	Plant growth	(Rosenberg et al., 2009)
Pea seedlings	–	Amoeba	Plant hormone production (auxins and related substance); Plant growth and biomass	(Nikoljuk, 1969)
Cress (*Lepidium sativum* L.)	Phyllosphere	*Acanthamoeba castellanii*	Plant hormone production (auxins); Shoot growth	(Krome et al., 2010)
	Rhizosphere (plant roots)		Plant hormone production (auxins); Root growth	
*Arabidopsis thaliana*	Phyllosphere Rhizosphere (plant roots)		Plant hormone production (auxins and cytokinin); Root growth	
Watercress seedlings	Rhizosphere (plant roots)	*Acanthamoeba castellanii*	Plant hormone production (auxin, indolyl‐3‐acetic acid (IAA)); Root growth and architecture	(Bonkowski & Brandt, 2002)

### Phyllosphere‐associated protists

Protists form key members of the plant microbiome and an external force shaping the plant microbiome assembly (Geisen et al., 2018; Gao et al., 2019), but the diversity and feedback of protists on phyllosphere microbiome remain surprisingly unknown. The occurrence of a protist strain *Colpoda cucullus* in leaves and stems in the 1970 s is one of early findings about the phyllosphere‐associated protists (Bamforth, 1973). Protists, especially the phylum Cecozoa consumers, have been recently identified in the model plant *Arabidopsis thaliana* (Sapp et al., 2018), sorghum (Sun et al., 2021a), grasses, legumes and forbs (Flues et al., 2017; Flues et al., 2018), with the ability to improve plant growth and biomass. The phyllosphere, a habitat of various phages, prokaryotes, protists, fungi, and visiting insects (e.g., bees, butterflies and herbivores), is supposed to regulated by their complex trophic interactions under direct impacts of environmental changes. Protists shape the community composition and activities of bacteria and fungi through selective predation (Bonkowski, 2004; Gao et al., 2019). Notably, the selective predation of protists triggers distinct bacterial strains to produce antimicrobials, such as 2,4‐diacetylphloroglucinol (DAPG) and pyrrolnitrin (Jousset et al., 2006), or violacein (Matz et al., 2004), which has been recorded in the interaction between one or a few model protist and bacterial species under *in vitro* conditions. Hence, protists may stimulate bacteria or fungi to excrete toxic metabolites to protect plants from air‐borne pathogens or herbivores.

Furthermore, protists can potentially select beneficial traits of microbes through (i) promoting phytohormone‐producing bacteria and ultimately enhancing plant fitness and development; and (ii) regulating the metabolic and functional profiles of bacterial community in the phyllosphere (Figure 1). Some first evidence about phytohormone stimulation of protists has been found in plant rhizosphere, and their beneficial effects on plant hormones in the phyllosphere are a fertile area to discover. Recent studies have indicated that bacterivorous amoebae promoted bacteria producing essential phytohormones (auxin and cytokinin) in the plant rhizosphere though protists alone cannot produce plant hormones (Bonkowski & Brandt, 2002; Krome et al., 2010). Flues et al. (2017) revealed that, through a shotgun metagenomic sequencing, the predation of leaf‐associated protists *Cercomonas* and *Paracercomonas* strains (Cercozoa) dramatically influenced the taxonomic composition and metabolic functions of leaf‐associated bacterial community under *in vitro* conditions, suggesting the strong regulation of protists on the activities and functions of bacteria in the phyllosphere. Many other representatives of leaf‐associated Cercozoan consumers (*Rhogostoma* spp.) were found to feed on fungi (here are yeasts) and algae in the phyllosphere of *A. thaliana*, and this grazing activity indicated crucial effects of protists on a wide range of microbes in the phyllosphere.

Plants are not passively benefited by microorganisms but may proactively use the strategy ‘cry for help’ to recruit beneficial microorganisms to protect themselves under the abiotic (e.g., drought or high temperature) and biotic stresses (e.g., pathogens or herbivores). The underlying mechanisms and recruited microorganisms of this strategy, however, are unclear and probably distinct across plant compartments. Strikingly, a board spectrum of bacteria and fungi (e.g., yeasts) inhabit the anthosphere (i.e., flowers and surrounding zones), especially nectar, pollen (Vannette et al., 2013; Schaeffer et al., 2017) and flower surface (Ushio et al., 2015; Arunkumar et al., 2019), which significantly influence flower‐pollinator interactions, plant reproduction and yield. Due to the diverse microbes transmitted from various sources, flowers are potentially dynamic hubs of microbes and pollinators. However, the diversity and roles of protists in the anthosphere are far from being fully elucidated. Moreover, endophytic protists colonize root and leaf and stem endosphere, where their interplay with plant hosts and other microbes can possibly influence plant hormones, defensive systems and nutrient translocation to every plant tissue. The stimulation of uptake and translocation of nitrogen from rhizosphere soils, plant roots to shoots by protists were reported in wheat plants (Clarholm, 1985; Henkes et al., 2018). Notably, the amoebae *Acanthamoeba castellanii* promoted the phytohormone production (auxins and cytokinin) of bacteria in the phyllosphere of cress (*Lepidium sativum* L.) and *A. thaliana* (Krome et al., 2010). Most recent studies have attempted to characterize the compositions of protists in the plant microbiome (Dumack et al., 2022; Sun et al., 2021a), hence further insights into the multitrophic interactions of protists with plants, microbes, air‐borne pathogens and insects in the phyllosphere are required.

### Rhizosphere‐associated protists

In contrast to other plant compartments, protists in the rhizosphere have received more attention with growing evidence for their crucial roles in (i) plant health and disease control (Xiong et al., 2020), (ii) nutrient cycling (Clarholm, 1985; Bonkowski, 2004), and (iii) plant hormones and growth (Bonkowski & Brandt, 2002). Many bacterial and fungal taxa are well‐known producers of antibiotics and toxic metabolites (Hutchings et al., 2019). The selective predation or even the presence of protists can trigger bacteria to produce specific antibiotics as weapons to kill or avoid protists through species‐specific response (Nguyen et al., 2020). For instance, *Pseudomonas fluorescens* strain SS101 and *Pseudomonas fluorescens* strain SBW25 produced antibiotics massetolide and viscosin, respectively, in response to the same bacterivorous amoeba *Naegleria americana* C1 (Mazzola et al., 2009; Song et al., 2015). Fungi also emit antimicrobial volatiles to inhibit the bacterial motility or growth upon bacterial–fungal interaction (Rybakova et al., 2017; Bruisson et al., 2020). However, there is a paucity of effects of protists on the antibiotic excretion of fungi. The antibiotics produced by bacteria and fungi are considered as a defensive mechanism to toxify not only protists but also other microbial competitors in natural habitats (Święciło, 2016; Cruz‐Loya et al., 2019). Through this effect, when plants ‘cry for help’ by sending signals via root exudates (volatiles, organic acids or others) under pathogen or pest attacks (Liu et al., 2019), protists may respond by recruiting antibiotic producers to produce antimicrobials to inhibit pathogens or pests for plant protection. However, this strategy of plants and their associations with protists are still elusive questions.

As primary microbial predators, protists can also directly consume bacterial and fungal pathogens. The consumptive effect of protists, typically protistan consumers, can cause fatality of a wide range of bacterial and fungal strains (Chakraborty et al., 1983; Dumack et al., 2016). In the rhizosphere of *A. thaliana*, the diversity and abundance of specific bacteria taxa, especially *Betaproteobacteria* and *Firmicutes*, were significantly decreased under the predation of soil amoeba *A. castellanii*. Bahroun et al. (2021) reported that bacterivorous protists alone and their synergistic interactions with bacteria reduced disease severity caused by a fungal pathogen *Fusarium solani* S55 and improved root length and plant growth of faba bean (*Vicia faba*) seedlings (Table 1). Recent studies have indicated important links of protists to soil‐borne disease control and plant health in the rhizosphere of tomatoes (Xiong et al., 2020), cucumber (Guo et al., 2021) and banana plants (Guo et al., 2022). In particular, numerous Cercozoan and Amoebozoan species can function as important indicators for the health of tomato plants. Guo et al. (2022) also revealed that the protistan consumer *Cercomonas lenta* strain ECO‐P‐01 substantially suppressed the density of the fungal pathogen *Fusarium oxysporum* and increased the disease‐suppressive bacteria *Bacillus* in the rhizosphere, and subsequently improved banana plant growth and yield. Hence, a comprehensive understanding of protists in the rhizosphere and other plant compartments will promote their applications in plant disease suppression.

Protists are also pivotal contributors to nutrient cycling in the rhizosphere (Table 1). Nutrients are temporarily locked up in rhizosphere bacterial and fungal biomass and can be translocated to protists as microbial feeders or unlocked by the protists' predation and eventually channelled to benefit plants, which is called ‘the microbial loop’ (Clarholm, 1985). Protists directly release nitrogen and carbon after prey digestion or form a symbiotic relationship with beneficial fungal or bacterial taxa in cycling essential nutrients (nitrogen, carbon, iron, silicon or phosphorous) (Geisen et al., 2018; Gao et al., 2019), enhancing soil nutrient input and fertility for nurturing plant growth and rhizo‐microbiome. The great contribution of protists to nutrient cycling has long been recognized since 1985, when Clarholm demonstrated the increasing nitrogen uptake to 75% by plants under the inoculation of protists. The presence of protists promoted plant phosphorus and calcium uptake and translocation to stems or needles, as well as modulated nutrient concentrations (nitrogen, phosphorus, carbon to nitrogen ratio (C/N ratio), calcium and magnesium) (Bonkowski et al., 2001). Consequently, this regulation of protists led to the improvement of root growth and architecture as well as biomass of different compartments (shoots, roots and needles) of spruce seedlings. A similar beneficial effect of protists was found in rice plants (*Oryza sativa* L.) (Henkes et al., 2018). Moreover, phototrophic protists contribute to carbon cycling as carbon fixers via photosynthesis (Schmidt et al., 2016), providing nonnegligible carbon and oxygen inputs to rhizosphere organisms and the basis for soil life, but their capacity for carbon sequestration is still unknown.

Notably, benefits of protists to plant nutrition are more efficient when forming symbiosis with other microbes, particularly arbuscular mycorrhizal fungi (AMF) that enhance plant nitrogen and phosphorus uptake. Protists might facilitate nutrient acquisition, mineralization and translocation of AMF (Zuccaro et al., 2014; Henkes et al., 2018), and promote the growth and activities of nitrifying bacteria and other bacteria (Bonkowski, 2004), suggesting intimate protist–microbe links in plant benefits. For instance, Bonkowski et al. (2001) indicated that the joint effects of protists and mycorrhiza significantly enhanced the phosphorous uptake from roots to stems, as well as affected rhizosphere microbes and essential plant nutrients (carbon, phosphorous and trace elements), which maximized the biomass of different spruce compartments (shoots, stems and needles). Protists, in rumen ecosystems, were detected to have positive links to archaea (Solomon et al., 2022), which are key players in the global nitrogen cycle (Hu et al., 2015). However, the contribution of archaea to plant hosts and protist‐archaea relationships in nutrient cycle is an intriguing unexplored topic. Upon nutrient shortage, beneficial protist–microbe interactions may be boosted by the plant strategy ‘cry for help’, and we cannot have a full understanding of protists' roles if ignoring their contributions.

Protists can also significantly influence plant hormones and development through regulating the community structure and activities of plant‐hormone producing rhizobacteria. Plant growth‐promoting phytohormones auxins (indolyl‐3‐acetic acid (IAA)) were found in the inoculation of the most studied model species *A. castellanii* in bacterial cultures (Nikoljuk, 1969) and rhizosphere of watercress seedlings (*L. sativum*) by modulating phytohormone‐producing bacteria or rhizobacterial community (Bonkowski & Brandt, 2002). While root systems are paramount apparatus to take up and allocate water and nutrients to every plant tissue for plant growth and environmental adaptation, protists, such as *A. castellanii*, can trigger phytohormone production (auxins and cytokinin) of bacteria, resulting in the enhancement of root growth and architecture and development of plants *L. sativum* and *A. thaliana*, more than bacteria standalone (Bonkowski & Brandt, 2002; Krome et al., 2010). Interestingly, the regulation on phytohormone‐producing bacteria strengthens root growth and architecture of many crops, including watercress, pea and cress (Table 1). Hence, it is evident that soil‐ or rhizosphere‐associated protists can significantly influence both the above‐ and below‐ground compartments of the plant hosts. While microbes are acknowledged as important hormone producers of plants (Nakano et al., 2022), more explorations of protists' roles in the inter‐organismal phytohormone networks between plant hosts, protists and other microbes are critical to deploy beneficial protists in improving plant immunity and development.

### Protists in bulk soils

In bulk soils, the diversity of protists is higher than that in the rhizosphere, root and litter (Ceja‐Navarro et al., 2021; Fiore‐Donno et al., 2022), which indicates that soil protists function as a ‘microbial seed bank’ for plant support and soil functions, as well as the selection of plants for protist communities. Moreover, protists can influence elemental cycles, soil fertility and soil microbiome by (i) steering the composition and activities of beneficial microorganisms (e.g., AMF or nitrifying microbes), (ii) excreting nitrogen or carbon sources after the predation and consumption of prey in bulk soils, and (iii) mediating the community composition and interactions of soil microbiome via facilitative, symbiotic or predatory relationships between protists and other microbes. The positive relationships between protists and bacteria have been identified in soil ecosystems (Nguyen et al., 2021), but further research is required to disentangle mechanisms for the interplay and roles of protists, fungi, bacteria, archaea, and viruses in plant‐associated microbiota. Beside the aforementioned benefits, parasitic protists have negative effects on plants, as pathogens have been more thoroughly characterized than neutral and beneficial protists (Dumack & Bonkowski, 2021). A large number of non‐pathogenic endophytic protists inhabit plant tissues and across rainforest soil ecosystems (Mahé et al., 2017), but their identity and functions on plant hosts remain unknown. Given their high abundance in natural habitats, we suppose that endophytic protists have unexplored benefits to plant hosts.

## FUTURE FRAMEWORK FOR UNRAVELLING THE ROLES OF PLANT‐ASSOCIATED PROTISTS IN PLANT HEALTH AND PRODUCTION IMPROVEMENT

**FIGURE 2:**
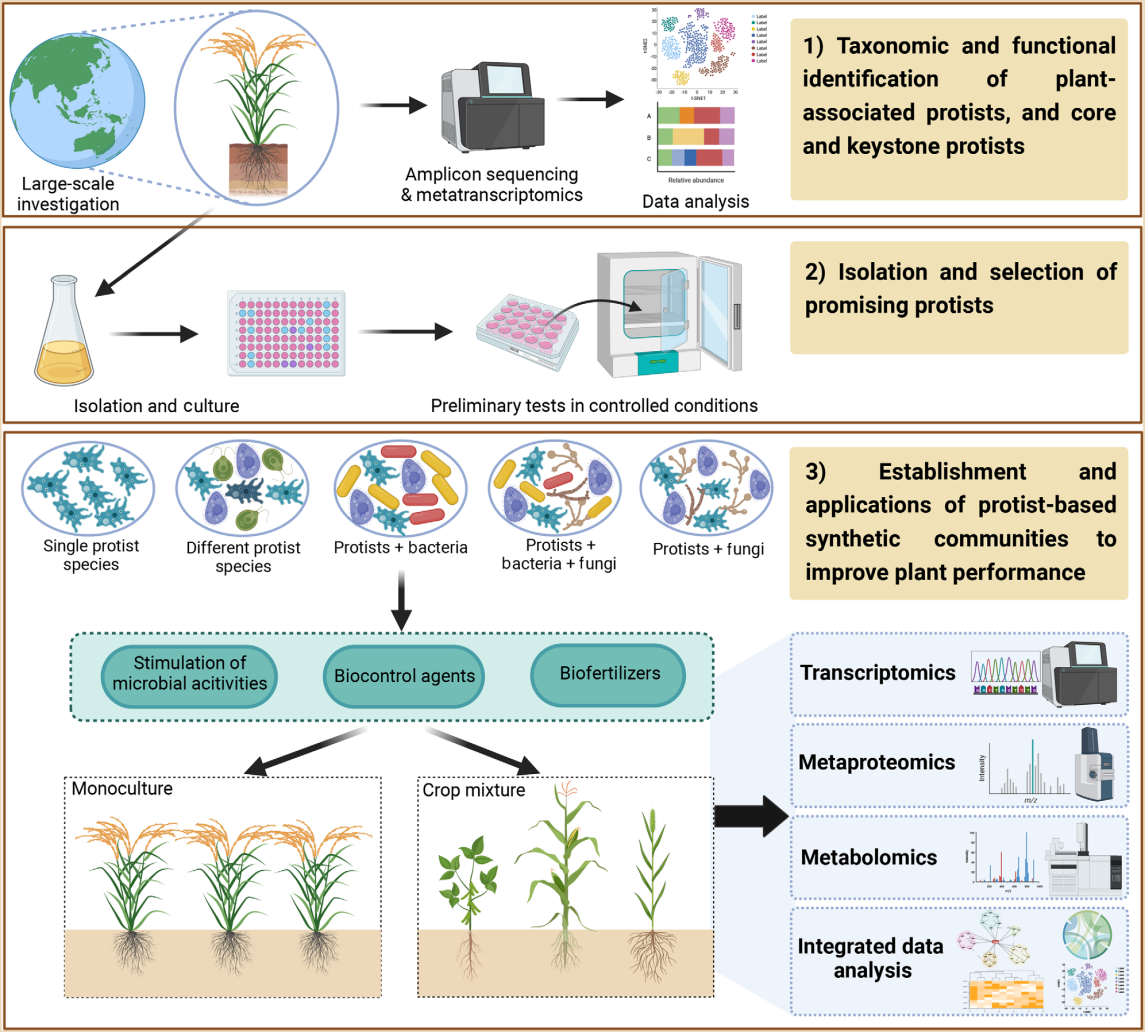
Proposed framework for future studies of plant‐associated protists and harnessing protist‐based products for improving crop production: (1) identification of key factors structuring the taxonomic and functional traits of plant‐associated protists and plant core protists in large‐scale investigations; (2) isolation and selection of potential plant beneficial protist candidates through data integration and preliminarily tests in short‐term controlled conditions; (3) establishment and applications of protist‐based synthetic communities (SynComs) to improve plant performance in monoculture or crop mixture. Different SynComs constituted by different inoculants: single protist species, different protist species, protists‐bacteria, protists‐bacteria‐fungi, and protists‐fungi. The integration of ‘omics’ techniques (i.e., metatranscriptomics, metaproteomics and metabolomics) with integrated data analysis characterizes cellular activities, functions and metabolites of protists, plant host and other organisms in the plant holobiont. The selection of crop species in this figure is just for illustration.

Protists alone or their interactions with other microbes are considered to play crucial roles in the plant holobiont. It is promising to develop protist‐based tools to enhance nutrient availability and plant growth as biofertilizers, to control plant disease infection and microbial functions as biocontrol agents, or to promote plant hormones and nutrient cycling activities and survival of plant beneficial microbes in modern agriculture. Compared to other plant microorganisms, the functions, signalling and feedbacks of protists in multi‐organismal (host–protist, protist–microbe, and protist–visiting insects) interactions with or without the infection of soil‐borne or air‐borne plant pathogens and pests are largely unexplored. A more comprehensive understanding of the molecular mechanisms and functions of plant–protist–microbe interactions will enable us to steer the activities and performance of microbes in the plant holobiont. Therefore, we propose and discuss future frameworks to generate a holistic view of plant‐associated protists and the manipulation and applications of protist‐based models in crop production, namely: (i) identification of key factors structuring the taxonomic and functional traits of plant‐associated protists as well as the core and keystone taxa of protists; (ii) isolation and selection of plant beneficial protists for various crops under different stresses; and (iii) establishment and applications of protist‐based synthetic communities (SynComs) to improve plant performance (Figure 2).

Firstly, the identification of key factors structuring the taxonomic and functional traits of plant‐associated protists is a crucial step. To date, most studies have characterized plant‐associated protists by conventional (microscopy‐based and direct counting) methods, quantitative PCR or amplicon sequencing. Identifying microbial eukaryotes with high throughput sequencing techniques, however, is not straight forward, since severe primer‐biases were identified in previous protist surveys (Lentendu et al., 2014; Hirakata et al., 2019). For instance, although many soils are known to be dominated by protists of the taxa Amoebozoa and Cercozoa, the primer‐based surveys constantly underestimate the importance of Amoebozoa (Bonkowski et al., 2019). Metatranscriptomics can overcome this issue as they do not rely on primers and, in accordance to what is found by morphological surveys, Amoebozoa may dominate in such datasets (Urich et al., 2008; Geisen et al., 2015). Furthermore, it is still difficult to estimate exact functioning of protists. Trait databases are helpful for the exploration of functioning in microbial eukaryotes (Dumack et al., 2020), but there is still a lack of a database covering all distinct protistan taxa. The characterization of protists in different plant compartments (including phyllosphere, leaf, stem and root endosphere, rhizosphere soil and bulk soil) in large‐scale field investigations is important to have full understandings about their taxonomic and functional diversity and community compositions for each plant species. The combination with co‐occurrence networks and statistical modellings will further disentangle the key drivers and principles shaping the protist community assembly and dynamics in plant microbiome, as well as build up a database of key protists that best predict plant performance parameters.

Many persistent and abundant members of a specific host found across wide‐range habitats constitute a core plant microbiota, which carry essential genes to support plant fitness as well as play crucial roles in maintaining multiple functions and stability of the host microbiome (Shade & Stopnisek, 2019). Core bacterial taxa, for example, members of the orders Rhizobiales and Pseudomonadales, are reported to benefit plant fitness, growth and resilience under stresses (Trivedi et al., 2020). Notably, keystone taxa of protists, highly associated members regardless of their abundance, deserve special attention because they crucially affect community structure and functions (Banerjee et al., 2018). Therefore, the determination of core and keystone taxa of protists for major crops across different regions, along with core and keystone taxa of bacteria and fungi (Banerjee et al., 2018; Trivedi et al., 2020), will leverage our capacity to manipulate plant microbial activities and design optimal SynCom models for maximizing growth and yields of specific crops. Crucial for this is a coupling of high throughput sequencing and metatranscriptomic approaches with subsequent culture attempts, first to identify core symbionts and then to provide them as a culture to research.

Secondly, to incorporate protists into the agrifood toolbox, it is paramount to establish a collection of plant beneficial protists for various crops under different stress conditions. It is promising to tailor the high‐throughput isolation approach which has proved to be effective in isolating bacterial strains from root microbiota (Zhang et al., 2021), to characterize and isolate protists from various plant tissues (e.g., leaf and stem endophytes). In the first selection step, the data integration of plant protists from large‐scale investigations with findings of the high‐throughput protist isolation will be a crucial reference for selection and nomination of promising protist species to establish protist‐based SynComs for improving plant performance. In the second selection step, the selected protist species, alone or in a subset of core and keystone protists, can be preliminarily tested for their capacity in performing desired functions, such as suppression of common fungal pathogens and resistance to abiotic stresses, in short‐term controlled laboratory conditions. Core and keystone taxa of protists conferring desired plant‐beneficial functions will be considered as key members of the protist‐based SynComs. However, other core and keystone taxa of protists, which do not have the desired features in the preliminary tests, should not be discarded because their performance may be boosted in facultative or antagonistic interactions with specific microbes.

Thirdly, protist isolates alone or combined with beneficial bacterial or fungal strains are used to construct different protist‐based synthetic communities to improve plant health and performance. These SynComs can mimic biological interactions (e.g., competition, predation or symbiosis) in natural settings, and the diversification of trophic interactions (e.g., bottom‐up and top‐down controls or trophic cascade) will boost microbes to produce crucial products (e.g., phytohormones, antibiotics and other compounds), and consequently stabilize the phytobiome and promote crop development. We propose to apply the protist‐based SynCom models for monoculture or mixture of plant species. Neighbouring crops in the plant mixture can increase interspecific interactions and functions of beneficial microbes, and plant uptake of essential resources (nutrients or water), with positive consequences for disease suppression and plant growth (Jing et al., 2022). All SynComs will be assessed for their efficacy in benefiting plant fitness and growth, nutrient cycling and uptake, disease controls and stress tolerance for each plant species. Some protist‐based products, for example, have hit the market and been applied in crop production, such as a protist species *Nosema Iocustae* as a biological control agent in over 90 species of grasshoppers, locusts, and crickets in the United States (https://www.gardeninsects.com/grasshopperbait.asp); and 19 biofertilizers developed from a mixture of beneficial protists, bacteria and fungi enhancing nutrients, plant growth and resilience for a variety of crops in Netherlands (https://ecostyle.nl/zoeken?query=protozoa).

In this step, the integration of multiple ‘omics’ techniques (including metatranscriptomics, metaproteomics and metabolomics) with machine learning and statistical modelling, rather than amplicon sequencing or one single method, will enable us to characterize the panoramic profile of cellular activities, functions, molecular signalling and metabolites of protists, plant host and other organisms in the plant holobiont. The metatranscriptomics elucidate the microbial identification, gene expression and functional profile of protists and other organisms, while metaproteomics (e.g., matrix‐assisted laser desorption‐ionization time of flight (TOF)/TOF‐mass spectrometry (MS)) is powerful to unravel protein identification, quantification and origin (Wang et al., 2011). Metabolomics can detect and quantify untargeted primary metabolites (e.g., organic acids, amino acids, and others), such as by gas chromatography (GC)‐MS, and secondary metabolites produced by plant hosts, protists and other associated microorganisms, such as by liquid chromatography (LC)‐high‐resolution MS (LC‐HRMS) (Weckwerth, 2010; Sumner et al., 2015). The application of machine learning and statistical modelling with transcriptomic, proteomic and metabolomic data can maximize our capacity to identify and predict compound composition, metabolic pathway, functional traits and activities of protists with the plant hosts and other organisms. For instance, the software METABOLIC is an advanced toolkit to profile metabolic and biogeochemical traits, and functional networks in microbial communities (Zhou et al., 2022). This integrated strategy will help us to explore and confirm the multifunctionality and benefits of plant‐associated protists to plant hosts and understand the complex trophic interactions within the plant–soil system in a holistic manner.

The development of protist‐based SynCom models as agrifood tools is potential to improve agricultural production. It is obvious that there will not be ‘one size fits all’ SynComs (Vorholt et al., 2017), hence the construction of protist‐based solutions should target species‐ or tissue‐specific SynComs for distinct plants at different developmental stages to optimize the efficacy in plant productivity, like commercial fertilizers or pesticides. Given the above‐mentioned contributions of protists to plants, we advocate for future efforts to target the development of beneficial protists as novel and sustainable biofertilizers for improving plant growth and productivity, biological control agents for enhancing pathogenic defence, and biological stimulation strategies for boosting microbial activities and plant‐promoting traits for plant health and performance. Biofertilizers are gaining interests across the agricultural sector, due to the recent rapid increase of fossil fuel price and fertilizer costs, the inoculation and formulation of protists into biofertilizers will be powerful to unlock natural nutrient sources or inorganic and organic fertilizers in soils. Nevertheless, the lack of sufficient knowledge about the roles of protists in soil ecology limits our ability to manage soil health for sustaining crop production. Future research on unravelling the functions and strategies of beneficial plant‐associated protists is necessary to enhance plant health and production, thus reducing the application of fungicide and pesticides.

## CONCLUDING REMARKS

Protists are key members of the plant‐associated microbiota. It is evident that their contributions alone or in combination with other microorganisms significantly benefit plants in not only a single but multiple aspects, such as plant nutrition, disease control, plant health and performance. The interplay between the hosts and associated protists in different plant and soil compartments is complex and still far from being fully elucidated. Therefore, there are calls to disentangle driving factors and key roles of protists in ecological processes and agricultural productivity, which can provide new insights into the manipulation and applications of beneficial protists as biofertilizers and other agricultural products in benefiting crop health and productivity and ultimately sustaining healthy agricultural systems. Although the protists' benefits to plants as bio‐fertilizers or biocontrol agents have been aware, we still have limitations on approaches studying the identity and functions of protists, as well as challenges in how to engineer efficient protist‐based SynComs and how to maintain their persistence and efficacy in crop production. The innovation of plant‐beneficial products from protists is a daunting task, but it will pay the ways to accelerate the development of protist‐based products and to innovate novel mobile molecular technologies to quickly assess and monitor the activities and community composition of the applied beneficial microbiome in smart‐farming systems and agricultural fields in the near future.

We also highlight some important questions about plant‐associated protists in the plant holobiont: (1) What are biochemical or molecular signals that protists recruit or pay partnerships with other plant‐associated microorganisms (e.g., bacteria or fungi) in benefiting plant hosts, as well as interact with insects (e.g., pollinators or ants) or herbivores? (2) What individuals or groups of protists are recruited by the plant hosts? (3) How do protists at different plant compartments respond to the strategy ‘cry for help’ of plants under biotic (pathogen or pest infection) or abiotic (e.g., low/high temperature, drought or salinity) stresses? (4) What are interactions between plant hosts and plant or soil microbiome under impacts of climate change? (5) Beside plant roots, do other plant tissues (e.g., leaf or stem) use a similar strategy ‘cry for help’ to interact with or recruit beneficial protists and other microorganisms for dealing with different stresses? (6) How can SynComs and other protist‐based tools be safely introduced and applied to recipient soils and crops? (7) How can we estimate and maintain the efficiency and persistence of protist‐based SynComs and other tools in enhancing plant growth and productivity in recipient soils and crops? The answer to these questions is a challenge but also a great opportunity to leverage our capacity to deploy plant‐associated microbiota to improve crop health and performance. No single method but the integrated advanced approaches can help us fully understand complex interactions in the plant holobiont.

## AUTHOR CONTRIBUTIONS

**Bao‐Anh Thi Nguyen:** Conceptualization; literature investigation; writing – original draft; writing – review and editing. **Kenneth Dumack:** writing – review and editing. **Pankaj Trivedi:** Writing – review and editing. **Zahra Islam:** Writing – review and editing. **Hang‐Wei Hu:** Conceptualization; funding acquisition; supervision; writing – review and editing.

## CONFLICT OF INTEREST

We declare that we have no competing interests.

## References

[emi16303-bib-0001] Arunkumar, N. , Rakesh, S. , Rajaram, K. , Kumar, N.R. & Durairajan, S.S.K. (2019) Anthosphere microbiome and their associated interactions at the aromatic interface. In: Plant microbe interface. Cham: Springer, pp. 309–324.

[emi16303-bib-0002] Bahroun, A. , Jousset, A. , Mrabet, M. , Mhamdi, R. & Mhadhbi, H. (2021) Protists modulate Fusarium root rot suppression by beneficial bacteria. Applied Soil Ecology, 168, 104158.

[emi16303-bib-0003] Bamforth, S.S. (1973) Population dynamics of soil and vegetation protozoa. American Zoologist, 13, 171–176.

[emi16303-bib-0004] Banerjee, S. , Schlaeppi, K. & van der Heijden, M.G. (2018) Keystone taxa as drivers of microbiome structure and functioning. Nature Reviews Microbiology, 16, 567–576. 10.1038/s41579-018-0024-1 29789680

[emi16303-bib-0005] Bonkowski, M. (2004) Protozoa and plant growth: the microbial loop in soil revisited. New Phytologist, 162, 617–631. 33873756 10.1111/j.1469-8137.2004.01066.x

[emi16303-bib-0006] Bonkowski, M. & Brandt, F. (2002) Do soil protozoa enhance plant growth by hormonal effects? Soil Biology and Biochemistry, 34, 1709–1715.

[emi16303-bib-0007] Bonkowski, M. , Griffiths, B. & Scrimgeour, C. (2000) Substrate heterogeneity and microfauna in soil organic ‘hotspots’ as determinants of nitrogen capture and growth of ryegrass. Applied Soil Ecology, 14, 37–53.

[emi16303-bib-0008] Bonkowski, M. , Jentschke, G. & Scheu, S. (2001) Contrasting effects of microbial partners in the rhizosphere: interactions between Norway Spruce seedlings (Picea abies Karst.), mycorrhiza (*Paxillus involutus* (Batsch) Fr.) and naked amoebae (protozoa). Applied Soil Ecology, 18, 193–204.

[emi16303-bib-0009] Bonkowski, M. , Dumack, K. & Fiore‐Donno, A.M. (2019) The protists in soil—a token of untold eukaryotic diversity. In: Modern soil microbiology. Cham: CRC Press, pp. 125–140.

[emi16303-bib-0010] Bruisson, S. , Berg, G. , Garbeva, P. & Weisskopf, L. (2020) Volatile interplay between microbes: friends and foes. In: Bacterial volatile compounds as mediators of airborne interactions. Singapore: Springer, pp. 215–235.

[emi16303-bib-0011] Ceja‐Navarro, J.A. , Wang, Y. , Ning, D. , Arellano, A. , Ramanculova, L. , Yuan, M.M. et al. (2021) Protist diversity and community complexity in the rhizosphere of switchgrass are dynamic as plants develop. Microbiome, 9, 1–18. 33910643 10.1186/s40168-021-01042-9 PMC8082632

[emi16303-bib-0012] Chakraborty, S. , Old, K. & Warcup, J. (1983) Amoebae from a take‐all suppressive soil which feed on *Gaeumannomyces graminis tritici* and other soil fungi. Soil Biology and Biochemistry, 15, 17–24.

[emi16303-bib-0013] Chen, Q.‐L. , Hu, H.‐W. , He, Z.‐Y. , Cui, L. , Zhu, Y.‐G. & He, J.‐Z. (2021) Potential of indigenous crop microbiomes for sustainable agriculture. Nature Food, 2, 233–240. 10.1038/s43016-021-00253-5 37118464

[emi16303-bib-0014] Clarholm, M. (1985) Interactions of bacteria, protozoa and plants leading to mineralization of soil nitrogen. Soil Biology and Biochemistry, 17, 181–187.

[emi16303-bib-0015] Cruz‐Loya, M. , Kang, T.M. , Lozano, N.A. , Watanabe, R. , Tekin, E. , Damoiseaux, R. et al. (2019) Stressor interaction networks suggest antibiotic resistance co‐opted from stress responses to temperature. The ISME Journal, 13, 12–23. 30171253 10.1038/s41396-018-0241-7 PMC6298959

[emi16303-bib-0016] Dumack, K. & Bonkowski, M. (2021) Protists in the plant microbiome: an untapped field of research. In: The plant microbiome. New York, NY: Springer, pp. 77–84. 10.1007/978-1-0716-1040-4_8 33161541

[emi16303-bib-0017] Dumack, K. , Baumann, C. & Bonkowski, M. (2016) A bowl with marbles: revision of the thecate amoeba genus *Lecythium* (Chlamydophryidae, Tectofilosida, Cercozoa, Rhizaria) including a description of four new species and an identification key. Protist, 167, 440–459. 27631274 10.1016/j.protis.2016.08.001

[emi16303-bib-0018] Dumack, K. , Fiore‐Donno, A.M. , Bass, D. & Bonkowski, M. (2020) Making sense of environmental sequencing data: ecologically important functional traits of the protistan groups Cercozoa and Endomyxa (Rhizaria). Molecular Ecology Resources, 20, 398–403. 31677344 10.1111/1755-0998.13112

[emi16303-bib-0019] Dumack, K. , Feng, K. , Flues, S. , Sapp, M. , Schreiter, S. , Grosch, R. et al. (2022) What drives the assembly of plant‐associated protist microbiomes? Investigating the effects of crop species, soil type and bacterial microbiomes. Protist, 173, 125913. 36257252 10.1016/j.protis.2022.125913

[emi16303-bib-0020] Fiore‐Donno, A.M. , Human, Z.R. , Štursová, M. , Mundra, S. , Morgado, L. , Kauserud, H. et al. (2022) Soil compartments (bulk soil, litter, root and rhizosphere) as main drivers of soil protistan communities distribution in forests with different nitrogen deposition. Soil Biology and Biochemistry, 168, 108628.

[emi16303-bib-0021] Flues, S. , Bass, D. & Bonkowski, M. (2017) Grazing of leaf‐associated Cercomonads (protists: Rhizaria: Cercozoa) structures bacterial community composition and function. Environmental Microbiology, 19, 3297–3309. 28618206 10.1111/1462-2920.13824

[emi16303-bib-0022] Flues, S. , Blokker, M. , Dumack, K. & Bonkowski, M. (2018) Diversity of Cercomonad species in the Phyllosphere and rhizosphere of different plant species with a description of *Neocercomonas epiphylla* (Cercozoa, Rhizaria) a leaf‐associated protist. Journal of Eukaryotic Microbiology, 65, 587–599. 29377417 10.1111/jeu.12503

[emi16303-bib-0023] Gao, Z. , Karlsson, I. , Geisen, S. , Kowalchuk, G. & Jousset, A. (2019) Protists: puppet masters of the rhizosphere microbiome. Trends in Plant Science, 24, 165–176. 30446306 10.1016/j.tplants.2018.10.011

[emi16303-bib-0024] Geisen, S. , Tveit, A.T. , Clark, I.M. , Richter, A. , Svenning, M.M. , Bonkowski, M. et al. (2015) Metatranscriptomic census of active protists in soils. The ISME Journal, 9, 2178–2190. 25822483 10.1038/ismej.2015.30 PMC4579471

[emi16303-bib-0025] Geisen, S. , Mitchell, E.A. , Adl, S. , Bonkowski, M. , Dunthorn, M. , Ekelund, F. et al. (2018) Soil protists: a fertile frontier in soil biology research. FEMS Microbiology Reviews, 42, 293–323. 29447350 10.1093/femsre/fuy006

[emi16303-bib-0026] Geisen, S. , Hu, S. & Veen, G. (2021) Protists as catalyzers of microbial litter breakdown and carbon cycling at different temperature regimes. The ISME Journal, 15, 618–621. 33005005 10.1038/s41396-020-00792-y PMC8027204

[emi16303-bib-0027] Guo, S. , Xiong, W. , Hang, X. , Gao, Z. , Jiao, Z. , Liu, H. et al. (2021) Protists as main indicators and determinants of plant performance. Microbiome, 9, 1–11. 33743825 10.1186/s40168-021-01025-w PMC7981826

[emi16303-bib-0028] Guo, S. , Tao, C. , Jousset, A. , Xiong, W. , Wang, Z. , Shen, Z. et al. (2022) Trophic interactions between predatory protists and pathogen‐suppressive bacteria impact plant health. The ISME Journal, 16, 1–12. 35461357 10.1038/s41396-022-01244-5 PMC9296445

[emi16303-bib-0029] Hassani, M. , Durán, P. & Hacquard, S. (2018) Microbial interactions within the plant holobiont. Microbiome, 6, 1–17. 29587885 10.1186/s40168-018-0445-0 PMC5870681

[emi16303-bib-0030] Henkes, G.J. , Kandeler, E. , Marhan, S. , Scheu, S. & Bonkowski, M. (2018) Interactions of mycorrhiza and protists in the rhizosphere systemically alter microbial community composition, plant shoot‐to‐root ratio and within‐root system nitrogen allocation. Frontiers in Environmental Science, 6, 117.

[emi16303-bib-0031] Herdler, S. , Kreuzer, K. , Scheu, S. & Bonkowski, M. (2008) Interactions between arbuscular mycorrhizal fungi (glomus intraradices, Glomeromycota) and amoebae (*Acanthamoeba castellanii*, protozoa) in the rhizosphere of rice (*Oryza sativa*). Soil Biology and Biochemistry, 40, 660–668.

[emi16303-bib-0032] Hirakata, Y. , Hatamoto, M. , Oshiki, M. , Watari, T. , Kuroda, K. , Araki, N. et al. (2019) Temporal variation of eukaryotic community structures in UASB reactor treating domestic sewage as revealed by 18S rRNA gene sequencing. Scientific Reports, 9, 1–11. 31484981 10.1038/s41598-019-49290-y PMC6726610

[emi16303-bib-0033] Hu, H.‐W. , Chen, D. & He, J.‐Z. (2015) Microbial regulation of terrestrial nitrous oxide formation: understanding the biological pathways for prediction of emission rates. FEMS Microbiology Reviews, 39, 729–749. 25934121 10.1093/femsre/fuv021

[emi16303-bib-0034] Hu, H.W. , Chen, Q.L. & He, J.Z. (2022) The end of hunger: fertilizers, microbes and plant productivity. Microbial Biotechnology, 15, 1050–1054. 34767687 10.1111/1751-7915.13973 PMC8966006

[emi16303-bib-0035] Hubbard, C.J. , Li, B. , McMinn, R. , Brock, M.T. , Maignien, L. , Ewers, B.E. et al. (2019) The effect of rhizosphere microbes outweighs host plant genetics in reducing insect herbivory. Molecular Ecology, 28, 1801–1811. 30582660 10.1111/mec.14989

[emi16303-bib-0036] Hutchings, M.I. , Truman, A.W. & Wilkinson, B. (2019) Antibiotics: past, present and future. Current Opinion in Microbiology, 51, 72–80. 31733401 10.1016/j.mib.2019.10.008

[emi16303-bib-0037] Jing, J. , Cong, W.‐F. & Bezemer, T.M. (2022) Legacies at work: plant–soil–microbiome interactions underpinning agricultural sustainability. Trends in Plant Science, 27, 781–792. 35701291 10.1016/j.tplants.2022.05.007

[emi16303-bib-0038] Jousset, A. , Lara, E. , Wall, L.G. & Valverde, C. (2006) Secondary metabolites help biocontrol strain *Pseudomonas fluorescens* CHA0 to escape protozoan grazing. Applied and Environmental Microbiology, 72, 7083–7090. 17088380 10.1128/AEM.00557-06 PMC1636139

[emi16303-bib-0040] Krome, K. , Rosenberg, K. , Bonkowski, M. & Scheu, S. (2009) Grazing of protozoa on rhizosphere bacteria alters growth and reproduction of *Arabidopsis thaliana* . Soil Biology and Biochemistry, 41, 1866–1873.

[emi16303-bib-0041] Krome, K. , Rosenberg, K. , Dickler, C. , Kreuzer, K. , Ludwig‐Müller, J. , Ullrich‐Eberius, C. et al. (2010) Soil bacteria and protozoa affect root branching via effects on the auxin and cytokinin balance in plants. Plant and Soil, 328, 191–201.

[emi16303-bib-0042] Leach, J.E. , Triplett, L.R. , Argueso, C.T. & Trivedi, P. (2017) Communication in the phytobiome. Cell, 169, 587–596. 28475891 10.1016/j.cell.2017.04.025

[emi16303-bib-0043] Lentendu, G. , Wubet, T. , Chatzinotas, A. , Wilhelm, C. , Buscot, F. & Schlegel, M. (2014) Effects of long‐term differential fertilization on eukaryotic microbial communities in an arable soil: a multiple barcoding approach. Molecular Ecology, 23, 3341–3355. 24888892 10.1111/mec.12819

[emi16303-bib-0044] Liu, H. , Macdonald, C.A. , Cook, J. , Anderson, I.C. & Singh, B.K. (2019) An ecological loop: host microbiomes across multitrophic interactions. Trends in Ecology & Evolution, 34, 1118–1130. 31422890 10.1016/j.tree.2019.07.011

[emi16303-bib-0045] Mahé, F. , de Vargas, C. , Bass, D. , Czech, L. , Stamatakis, A. , Lara, E. et al. (2017) Parasites dominate hyperdiverse soil protist communities in neotropical rainforests. Nature Ecology & Evolution, 1, 1–8. 28812652 10.1038/s41559-017-0091

[emi16303-bib-0046] Matz, C. , Deines, P. , Boenigk, J. , Arndt, H. , Eberl, L. , Kjelleberg, S. et al. (2004) Impact of violacein‐producing bacteria on survival and feeding of bacterivorous nanoflagellates. Applied and Environmental Microbiology, 70, 1593–1599. 15006783 10.1128/AEM.70.3.1593-1599.2004 PMC368400

[emi16303-bib-0047] Mazzola, M. , De Bruijn, I. , Cohen, M.F. & Raaijmakers, J.M. (2009) Protozoan‐induced regulation of cyclic lipopeptide biosynthesis is an effective predation defense mechanism for *Pseudomonas fluorescens* . Applied and Environmental Microbiology, 75, 6804–6811. 19717630 10.1128/AEM.01272-09 PMC2772446

[emi16303-bib-0048] Nakano, M. , Omae, N. & Tsuda, K. (2022) Inter‐organismal phytohormone networks in plant–microbe interactions. Current Opinion in Plant Biology, 68, 102258. 35820321 10.1016/j.pbi.2022.102258

[emi16303-bib-0049] Nguyen, B.‐A.T. , Chen, Q.‐L. , He, J.‐Z. & Hu, H.‐W. (2020) Microbial regulation of natural antibiotic resistance: understanding the protist–bacteria interactions for evolution of soil resistome. Science of the Total Environment, 705, 135882. 31818598 10.1016/j.scitotenv.2019.135882

[emi16303-bib-0050] Nguyen, B.‐A.T. , Chen, Q.‐L. , Yan, Z.‐Z. , Li, C. , He, J.‐Z. & Hu, H.‐W. (2021) Distinct factors drive the diversity and composition of protistan consumers and phototrophs in natural soil ecosystems. Soil Biology and Biochemistry, 160, 108317.

[emi16303-bib-0051] Nikoljuk, V. (1969) Some aspects of the study of soil protozoa. Acta Protozoologica, 7, 1–37.

[emi16303-bib-0052] Rosenberg, K. , Bertaux, J. , Krome, K. , Hartmann, A. , Scheu, S. & Bonkowski, M. (2009) Soil amoebae rapidly change bacterial community composition in the rhizosphere of *Arabidopsis thaliana* . The ISME Journal, 3, 675–684. 19242534 10.1038/ismej.2009.11

[emi16303-bib-0053] Rybakova, D. , Rack‐Wetzlinger, U. , Cernava, T. , Schaefer, A. , Schmuck, M. & Berg, G. (2017) Aerial warfare: a volatile dialogue between the plant pathogen verticillium longisporum and its antagonist *Paenibacillus polymyxa* . Frontiers in Plant Science, 8, 1294. 28798756 10.3389/fpls.2017.01294 PMC5529406

[emi16303-bib-0054] Sapp, M. , Ploch, S. , Fiore‐Donno, A.M. , Bonkowski, M. & Rose, L.E. (2018) Protists are an integral part of the *Arabidopsis thaliana* microbiome. Environmental Microbiology, 20, 30–43. 28967236 10.1111/1462-2920.13941

[emi16303-bib-0055] Schaeffer, R.N. , Mei, Y.Z. , Andicoechea, J. , Manson, J.S. & Irwin, R.E. (2017) Consequences of a nectar yeast for pollinator preference and performance. Functional Ecology, 31, 613–621.

[emi16303-bib-0056] Schmidt, O. , Dyckmans, J. & Schrader, S. (2016) Photoautotrophic microorganisms as a carbon source for temperate soil invertebrates. Biology Letters, 12, 20150646. 26740559 10.1098/rsbl.2015.0646 PMC4785913

[emi16303-bib-0057] Shade, A. & Stopnisek, N. (2019) Abundance‐occupancy distributions to prioritize plant core microbiome membership. Current Opinion in Microbiology, 49, 50–58. 31715441 10.1016/j.mib.2019.09.008

[emi16303-bib-0058] Solomon, R. , Wein, T. , Levy, B. , Eshed, S. , Dror, R. , Reiss, V. et al. (2022) Protozoa populations are ecosystem engineers that shape prokaryotic community structure and function of the rumen microbial ecosystem. The ISME Journal, 16, 1187–1197. 34887549 10.1038/s41396-021-01170-y PMC8941083

[emi16303-bib-0059] Song, C. , Mazzola, M. , Cheng, X. , Oetjen, J. , Alexandrov, T. , Dorrestein, P. et al. (2015) Molecular and chemical dialogues in bacteria–protozoa interactions. Scientific Reports, 5, 1–13. 10.1038/srep12837 PMC4542665 26246193

[emi16303-bib-0060] Sumner, L.W. , Lei, Z. , Nikolau, B.J. & Saito, K. (2015) Modern plant metabolomics: advanced natural product gene discoveries, improved technologies, and future prospects. Natural Product Reports, 32, 212–229. 25342293 10.1039/c4np00072b

[emi16303-bib-0061] Sun, A. , Jiao, X.Y. , Chen, Q. , Trivedi, P. , Li, Z. , Li, F. et al. (2021a) Fertilization alters protistan consumers and parasites in crop‐associated microbiomes. Environmental Microbiology, 23, 2169–2183. 33400366 10.1111/1462-2920.15385

[emi16303-bib-0062] Sun, A. , Jiao, X.Y. , Chen, Q. , Wu, A. , Zheng, Y. , Lin, Y.X. et al. (2021b) Microbial communities in crop phyllosphere and root endosphere are more resistant than soil microbiota to fertilization. Soil Biology and Biochemistry, 153, 108113.

[emi16303-bib-0063] Święciło, A. (2016) Cross‐stress resistance in Saccharomyces cerevisiae yeast—new insight into an old phenomenon. Cell Stress and Chaperones, 21, 187–200. 26825800 10.1007/s12192-016-0667-7 PMC4786536

[emi16303-bib-0064] Trivedi, P. , Leach, J.E. , Tringe, S.G. , Sa, T. & Singh, B.K. (2020) Plant–microbiome interactions: from community assembly to plant health. Nature Reviews Microbiology, 18, 607–621. 32788714 10.1038/s41579-020-0412-1

[emi16303-bib-0065] Urich, T. , Lanzén, A. , Qi, J. , Huson, D.H. , Schleper, C. & Schuster, S.C. (2008) Simultaneous assessment of soil microbial community structure and function through analysis of the meta‐transcriptome. PLoS One, 3, e2527. 18575584 10.1371/journal.pone.0002527 PMC2424134

[emi16303-bib-0066] Ushio, M. , Yamasaki, E. , Takasu, H. , Nagano, A.J. , Fujinaga, S. , Honjo, M.N. et al. (2015) Microbial communities on flower surfaces act as signatures of pollinator visitation. Scientific Reports, 5, 1–7. 10.1038/srep08695 PMC4346974 25733079

[emi16303-bib-0067] Vannette, R.L. , Gauthier, M.‐P.L. & Fukami, T. (2013) Nectar bacteria, but not yeast, weaken a plant–pollinator mutualism. Proceedings of the Royal Society B: Biological Sciences, 280, 20122601. 10.1098/rspb.2012.2601 PMC3574316 23222453

[emi16303-bib-0068] Vorholt, J.A. , Vogel, C. , Carlström, C.I. & Müller, D.B. (2017) Establishing causality: opportunities of synthetic communities for plant microbiome research. Cell Host & Microbe, 22, 142–155. 28799900 10.1016/j.chom.2017.07.004

[emi16303-bib-0069] Wang, H.‐B. , Zhang, Z.‐X. , Li, H. , He, H.‐B. , Fang, C.‐X. , Zhang, A.‐J. et al. (2011) Characterization of metaproteomics in crop rhizospheric soil. Journal of Proteome Research, 10, 932–940. 21142081 10.1021/pr100981r

[emi16303-bib-0070] Weckwerth, W. (2010) Metabolomics: an integral technique in systems biology. Bioanalysis, 2, 829–836. 21083277 10.4155/bio.09.192

[emi16303-bib-0071] Xiong, W. , Song, Y. , Yang, K. , Gu, Y. , Wei, Z. , Kowalchuk, G.A. et al. (2020) Rhizosphere protists are key determinants of plant health. Microbiome, 8, 1–9. 32127034 10.1186/s40168-020-00799-9 PMC7055055

[emi16303-bib-0072] Zhang, J. , Liu, Y.‐X. , Guo, X. , Qin, Y. , Garrido‐Oter, R. , Schulze‐Lefert, P. et al. (2021) High‐throughput cultivation and identification of bacteria from the plant root microbiota. Nature Protocols, 16, 988–1012. 33442053 10.1038/s41596-020-00444-7

[emi16303-bib-0073] Zhou, Z. , Tran, P.Q. , Breister, A.M. , Liu, Y. , Kieft, K. , Cowley, E.S. et al. (2022) METABOLIC: high‐throughput profiling of microbial genomes for functional traits, metabolism, biogeochemistry, and community‐scale functional networks. Microbiome, 10, 1–22. 35172890 10.1186/s40168-021-01213-8 PMC8851854

[emi16303-bib-0074] Zuccaro, A. , Lahrmann, U. & Langen, G. (2014) Broad compatibility in fungal root symbioses. Current Opinion in Plant Biology, 20, 135–145. 24929298 10.1016/j.pbi.2014.05.013

